# Diagnostic accuracy of SPECT, PET, and MRS for primary central nervous system lymphoma in HIV patients: A systematic review and meta-analysis

**DOI:** 10.1097/MD.0000000000006676

**Published:** 2017-05-12

**Authors:** Mo Yang, James Sun, Harrison X. Bai, Yongguang Tao, Xiangqi Tang, Lisa J. States, Zishu Zhang, Jianhua Zhou, Michael D. Farwell, Paul Zhang, Bo Xiao, Li Yang

**Affiliations:** a Department of Neurology, The Second Xiangya Hospital, Changsha City, Hunan Province, China; b Department of Radiology, Hospital of the University of Pennsylvania, Philadelphia, PA, USA; c Cancer Research Institute, Central South University, Changsha City, Hunan Province, China; d Department of Radiology, Children's Hospital of Philadelphia, Philadelphia, PA, USA; e Department of Radiology, The Second Xiangya Hospital; f Department of Pathology, The First Xiangya Hospital, Changsha City, Hunan Province, China; g Department of Pathology, Hospital of the University of Pennsylvania, Philadelphia, PA, USA; h Department of Neurology, The First Xiangya Hospital, Changsha City, Hunan Province, China.

**Keywords:** HIV, MRS, PCNSL, PET, SPECT

## Abstract

**Background:**

We performed a systematic review and meta-analysis to assess the roles of SPECT, PET, and MRS in distinguishing primary central nervous system lymphoma (PCNSL) from other focal brain lesions (FBLs) in human immunodeficiency virus (HIV)-infected patients.

**Methods:**

PubMed, Scopus, and Medline were systematically searched for eligible studies from 1980 to 2016. Two authors extracted characteristics of patients and their lesions using predefined criteria.

**Results:**

Eighteen studies on SPECT containing 667 patients, 6 studies on PET containing 108 patients, and 3 studies on MRS containing 96 patients were included. SPECT had a pooled sensitivity of 0.92 (95% CI: 0.85–0.96) and specificity of 0.84 (95% CI: 0.74–0.90) in differentiating PCNSL from other FBLs. For the 6 studies that used only pathology and/or serology as the gold standard, the pooled sensitivity was 0.85 (95% CI: 0.72–0.97) and the pooled specificity was 0.73 (95% CI: 0.54–0.92).

**Conclusion:**

SPECT has good diagnostic accuracy for discriminating PCNSL from other FBL-causing disorders in HIV patients. However, the actual sensitivity and specificity of SPECT may be lower than expected if only pathology and/or serology was used as the gold standard. PET may be superior but has less supporting clinical data and is more expensive.

## Abstract 2

Supplemental Digital Content is available in the text

## 1 Introduction

Neurological complications affect 40% to 80% of human immunodeficiency virus (HIV)-infected patients.^[1]^ CNS-related pathology mainly results from opportunistic infections such as toxoplasmosis, cytomegalovirus, and progressive multifocal leukoencephalopathy.^[2]^ Primary central nervous system lymphoma (PCNSL) is also an important etiology of focal brain lesions (FBLs) in HIV-infected individuals with a reported incidence of 2% to 6%, at least 1000 times higher than that in the general population.^[3]^ Despite advances in new treatment strategies, the median survival of HIV patients with PCNSL not receiving radiation or other targeted therapies immediately after the onset of clinical symptoms is 1 month.^[4]^ Due to the rapid progression of the disease, a fast and reliable diagnosis is essential.

It is often difficult to differentiate PCNSL from other FBLs, particularly from toxoplasmosis in HIV patients. The 2 diseases share similar clinical symptoms and demonstrate nearly identical imaging features on routine CT and MRI.^[5–10]^ In these patients, it is standard practice to begin empirical antitoxoplasmosis treatment.^[11]^ Brain biopsy is usually performed when treatments fail to lead to clinical and radiological improvements. Brain biopsy, despite being the current diagnostic gold standard, is invasive and has high morbidity.^[12]^ Over the past few decades, noninvasive functional nuclear imaging modalities such as single-photon emission computed tomography (SPECT) and positron emission tomography (PET) have been used to detect PCNSL based on physiologic differences between lymphoma and infectious lesions. Several studies have shown high diagnostic accuracy of both SPECT^[10,13–17]^ and PET,^[18–23]^ but a few other studies have cast doubt on the utility of SPECT.^[5,24,25]^ Advanced MRI techniques, including apparent diffusion coefficient (ADC) ratios,^[26,27]^ regional cerebral blood volume (rCBV),^[28]^ and magnetic resonance spectroscopy (MRS),^[29–35]^ have demonstrated promise in differentiating PCNSL from other FBLs, but the results are variable. Due to the small number of studies and limited cohort sizes of existing studies, it is challenging to create guidelines for clinical practice based on published literature.

The main aim of this study is to perform a systematic review and meta-analysis of the published studies to assess the diagnostic accuracy of SPECT, PET, and MRS in differentiating PCNSL from other FBLs in HIV patients.

## 2 Methods

No institutional review board approval was required for this study. Preferred Reporting Items for Systematic Reviews and Meta-Analyses (PRISMA) guidelines were followed.^[36]^ The electronic databases used were PubMed, Scopus, and Medline. The keywords used were a combination of “HIV” or “AIDS,” “lymphoma,” “brain,” “SPECT” or “PET” or “MRI,” “toxoplasmosis,” and their variations. Reference lists of the included papers were reviewed to identify potential papers. Only studies published from 1980 to 2016 in English were included.

Two authors (MY and HXB) independently examined the titles and abstracts or full texts when necessary to determine eligible studies. In case of disagreement or uncertainty, a consensus was reached by consulting with a third author (LY). Inclusion criteria were: All patients included in the study were infected with HIV; All patients had FBLs on MRI and/or CT before SPECT, PET, or MRS was performed; The study contained data that could be converted to analyze diagnostic accuracy (ie, 2 × 2 table); and Final diagnosis was clearly stated in the paper. Exclusion criteria were: Cohort size was too small (<10); The patient cohort in the study overlapped with that in a previous study; The study focused on imaging modalities other than SPECT, PET, or MRS; and The study was in the format of letters, comments, case reports, or personal communications.

Two authors (MY and HBX) extracted data together from the 1st 5 articles and then individually for the rest of the articles. The variables extracted included: characteristics of the studies such as name of the first author, year of publication, country in which the study was conducted, study design (retrospective or prospective), and study size; patient characteristics such as age, gender, and final diagnoses of the nonlymphoma group; lesion characteristics such as the number of lesions in the brain and lesion size; imaging techniques such as modality and tracer used; analytical method (visual evaluation vs quantitative analysis); reference standard (pathological or serological diagnosis vs clinical follow-up); and average follow-up time of patients.

Two authors (MY and HBX) assessed the methodological quality of the selected studies using Quality Assessment of Diagnostic Accuracy Studies (QUADAS)-2. Discrepancies were resolved by consensus meetings in a panel including a 3rd author (LY). The risk of bias was analyzed in 4 domains: patient selection, index test, reference standard, and flow and timing. Applicability was assessed in the following 3 domains: patient selection, index test, and reference standard. Signaling questions were applied to determine the risk of bias and applicability.

We included prospective and retrospective studies of HIV patients with FBLs who had SPECT, PET, or MRS scans. We calculated the pooled sensitivity and specificity, likelihood ratio (LR), and the area under the receiver-operating characteristic curve (AUC). Hierarchical summary receiver-operating characteristic (HSROC) curve was constructed for SPECT. A 2-sided *P* value less than .05 was considered statistically significant. The nonthreshold effect that contributed to heterogeneity was assessed by chi-square (χ^2^) and inconsistency index (*I*^2^) statistics. If *I*^2^ > 50%, a random-effects model was used, and a fixed-effects model was used otherwise. Publication bias was evaluated by the Deek test for funnel plot asymmetry. Sensitivity analysis was conducted using the leave-one-out approach. In case of significant heterogeneity, subgroup analysis via meta-regression was performed to explore a potential source of heterogeneity by calculating the *I*^2^ statistics. The covariates investigated included study design, study size, geographical region, tracer applied, reference standard, analytical method, and percent of nonlymphoma final diagnoses which were toxoplasma encephalitis (TE). All analyses were performed using StataIC 14 (StataCorp, College Station, TX).

## 3 Results

**Figure 1:**
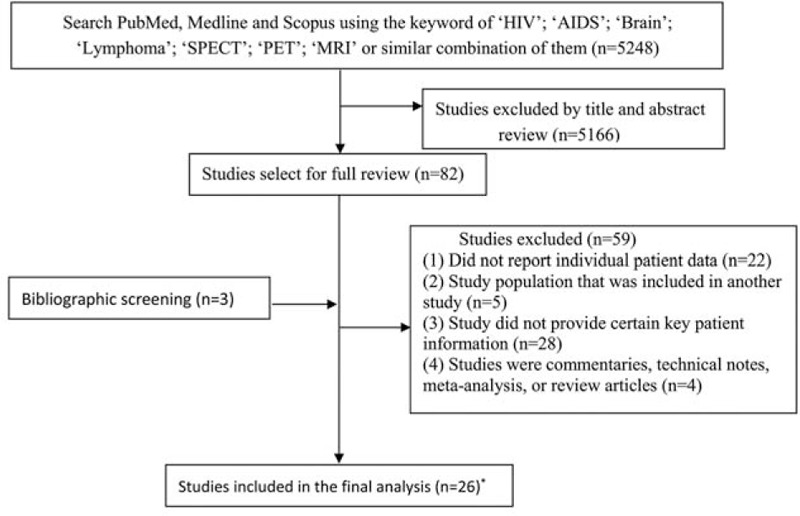
Flow chart for the identification of eligible studies. ∗Westwood et al studied both PET and MRS. MRS = magnetic resonance spectroscopy, PET = positron emission tomography.

We screened 5248 articles initially for potentially eligible studies (Fig. 1). Per inclusion criteria, 82 studies remained. After full review of the 82 papers, 59 studies were excluded. We included 3 additional studies by reviewing the reference lists of the 82 papers. A total of 26 studies were included in the final analysis: 18 studies on SPECT containing 667 patients, 6 studies on PET containing 108 patients, and 3 studies on MRS containing 96 patients. One study included both PET and MRS.^[37]^

The included studies were published between 1994 and 2016. Thirteen studies were from the US, 11 were from Europe, and 2 were from Asia. The average age of all included patients was 36.9 (range: 16–79). For SPECT studies, Thallium-201 was used as the only tracer in 14 studies and Technetium-99m sestamibi as the only tracer in 1. Three studies used a combination of different tracers, which were counted separately in our meta-analysis. For PET studies, all 6 studies used 18F-fluorodeoxyglucose as the only tracer. For SPECT, 5 studies used only pathology and/or serology as the reference standard for the final diagnosis, while 13 studies used clinical follow-up as the reference standard in addition to pathology and/or serology. For PET and MRS, all 6 PET studies and 3 MRS studies used a combination of pathology, serology, and clinical follow-up as the reference standard. Among the 17 studies on SPECT which provided information on method of analysis, quantitative analysis was performed in 10 studies, while qualitative analysis was applied in 7 studies. For PET, quantitative analysis was performed in 3 studies, while visual inspection was used in the other 3 studies. For MRS, all 3 studies used quantitative analysis. In all 3 MRS studies, NAA/Cr, NA/Cr, Cho/Cr, and NAA/Cho ratios were used as parameters to estimate the diagnostic accuracy of MRS. Among all studies, only 3 reported mean single lesion size.^[14,24,38]^ Shyam Babu et al separated all lesions into those larger than 1 cm and smaller than 1 cm.^[5]^ Characteristics of included studies on SPECT, PET, and MRS are summarized in Supplemental Tables 1 to 3.

Seven domains were assessed regarding bias and applicability for each paper. Out of the 182 domains in total including all papers, 23 domains were determined as high risk (15 for risk of bias and 8 for applicability concerns). Therefore, the overall quality was acceptable. We found that the bias stemmed mainly from the index test (n = 6), flow and timing (n = 6), and patient selection (n = 11) domains. The quality assessment of the 26 included papers using QUADAS-2 is shown in Supplemental Table 4.

**Figure 2:**
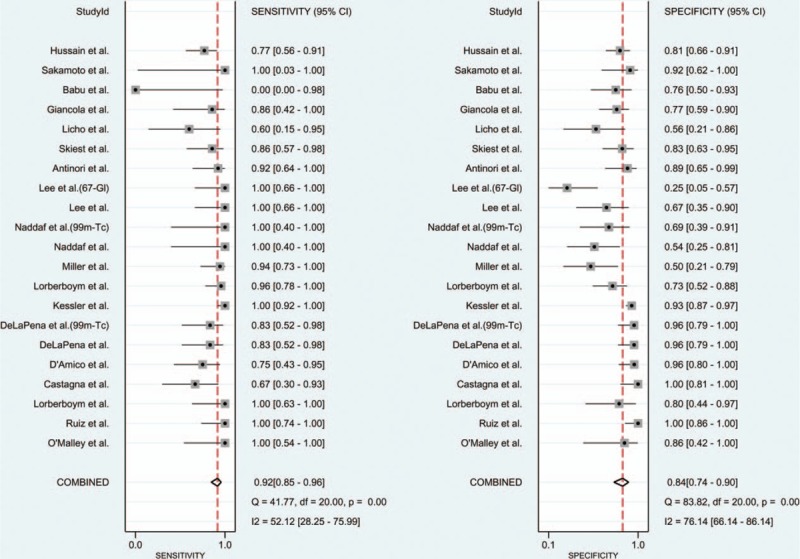
Forest plot of sensitivity and specificity for SPECT. All tracers used were thallium-201 chlorideunless specifically labeled. 67-Gl = gallium-67, 99m-Tc = technetium sestamibi, SPECT = single-photon emission computed tomography.

**Figure 3:**
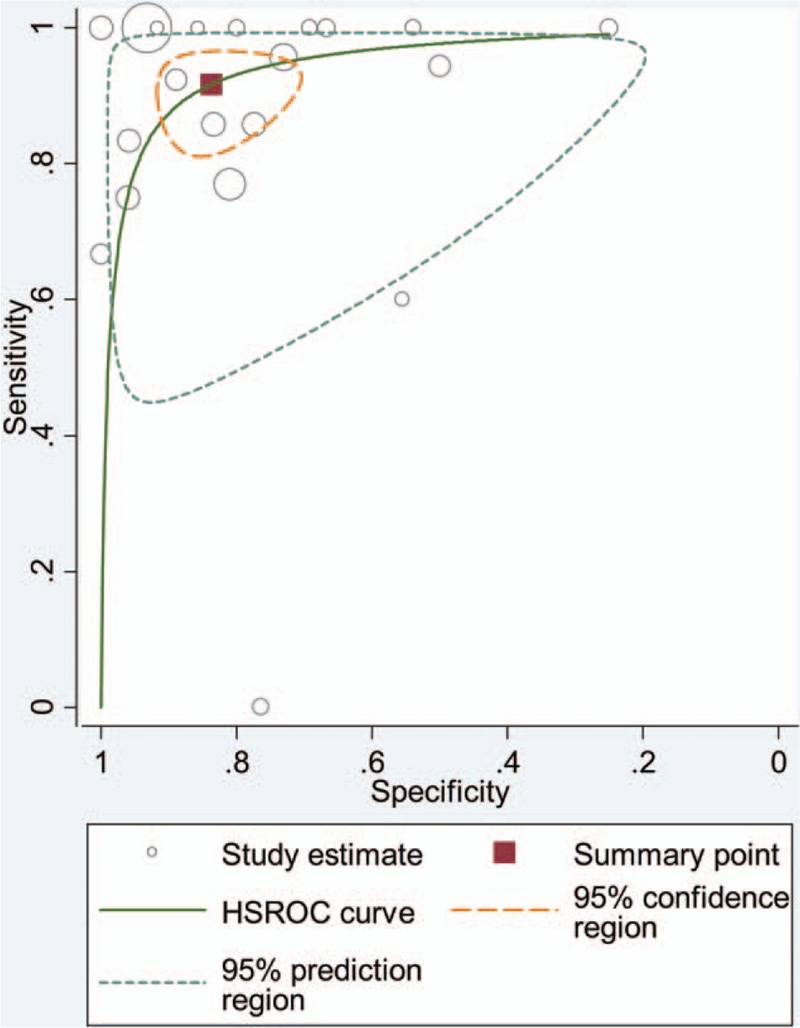
HSROC curve of SPECT for differentiating lymphoma from nonlymphoma FBLs in HIV patients. FBL = focal brain lesion, HIV = human immunodeficiency virus, HSROC = hierarchical summary receiver-operating characteristic, SPECT = single-photon emission computed tomography.

The forest plot of sensitivity and specificity for individual SPECT studies is shown in Fig. 2. The *I*^2^ values for sensitivity and specificity of the 18 included studies were 52.1 (95% CI: 28.3–76.0) and 76.1 (95% CI: 66.1–86.1), respectively. A random-effect model was used to calculate pooled sensitivity and specificity, which were 0.92 (95% CI: 0.85–0.96) and 0.84 (95% CI: 0.74–0.90), respectively (Fig. 2). The overall LR+ and LR− for SPECT were 5.6 (95% CI: 3.5–9.0) and 0.10 (95% CI: 0.05–0.19). The HSROC curve is shown in Fig. 3 with an area under curve (AUC) of 0.95. Pooled diagnostic odds ratio (DOR) of the 18 studies was 57 (95% CI: 25–131).

All 6 studies had a sensitivity of 100%. Regarding specificity, 4 out of 6 studies had a specificity of 100%, while the study by Pierce et al^[22]^ reported a specificity of 91% and the study by Heald et al^[18]^ reported a specificity of 75%. Further meta-analysis of PET studies could not be performed because no false negatives were reported for any of the 6 studies (ie, value of zero in 2 × 2 contingency table).

Three studies on MRS reported sensitivity of 0.71 (95% CI: 0.29–0.96), 0.50 (95% CI: 0.01–0.99), and 1.00 (95% CI: 0.63–1.00). The specificities were 0.83 (95% CI: 0.70–0.92), 0.60 (95% CI: 0.15–0.95), and 0.27 (95% CI: 0.08–0.55). Further meta-analysis could not be performed due to limited number of eligible studies.

Bivariate meta-regression analysis was performed to detect the source of heterogeneity. We found that the analytical method used and reference standard contributed to the heterogeneity, while geography, study design, study size, tracer used, and proportion of TE in nonlymphoma cases did not contribute significantly to heterogeneity. The pooled sensitivity of studies using quantitative analysis was higher than that based on studies using visual inspection (0.94 vs 0.86). Both sensitivity (0.94 vs 0.85) and specificity (0.87 vs 0.73) in studies including follow-up as reference standard in addition to pathology and/or serology were higher than those that used only pathology and/or serology as the gold standard. The results of the subgroup meta-regression analysis for SPECT are summarized in Table 1.

**Table 1:**
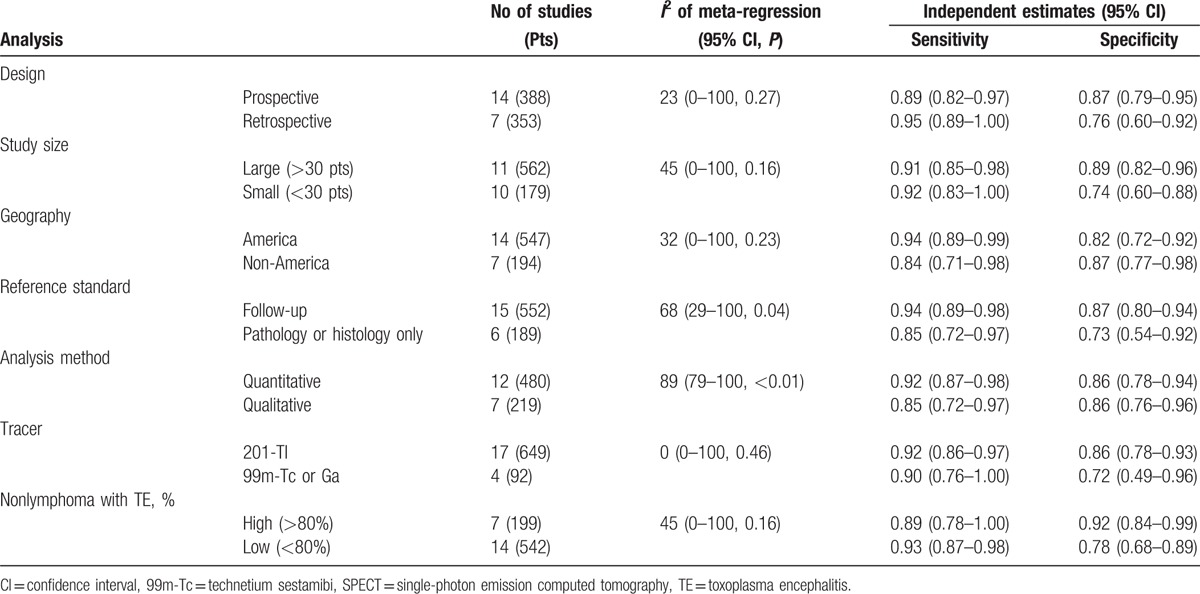
Meta-regression analyses and diagnostic performance of SPECT in subgroups of study characteristics.

Sensitivity analysis using the leave-one-out approach demonstrated that the direction and magnitude of estimates were not influenced by any 1 study removed for SPECT (Supplemental Table 5). Deek funnel plot and regression test of asymmetry demonstrated a publication bias (*P* = 0.001) (Supplemental Fig. 1).

## 4 Discussion

Since the introduction of highly active antiretroviral therapy (HAART) in 1996, the epidemiology of CNS disease among HIV patients has been altered.^[39]^ HAART reduces plasma viral load and thus significantly decreases the incidence of opportunistic infections such as toxoplasmosis and multifocal leukoencephalopathy in immunocompromised patients. However, the incidence of HIV-related lymphoma has been on the rise.^[40]^ The exact pathogenic mechanism of PCNSL is not well understood, but the B-cell malignancies are consistently associated with Epstein–Barr virus. Lymphomagenesis typically occurs late in the course of HIV infection.^[3]^

In the post-HAART era, a correct early diagnosis of brain lymphoma is essential for HIV patients, since lymphoma is managed differently from toxoplasmosis and other opportunistic infections.^[41]^ Unfortunately, brain biopsy is invasive while conventional imaging modalities have limited diagnostic accuracy.^[6,12]^ Functional nuclear imaging modalities such as SPECT and PET have become increasingly popular diagnostic tools, as they provide images with high spatiotemporal resolution to evaluate perfusion or metabolism. SPECT relies on gamma ray emission from uptake of radiotracers such as Tc-99m, while PET utilizes positron-emitting radiotracers such as ^18^F-fluorodeoxyglucose.^[42]^ MRS aims to query the biochemical composition of tissue by using the same ^1^H signals recognized by conventional MRI to determine relative concentrations of target brain metabolites such as N-acetylaspartate, choline, and creatine.^[43]^ A generally accepted understanding of the use of SPECT, PET, and advanced MRI techniques to diagnose brain lymphoma is currently lacking due to the small number of published studies and limited cohort size. Thus, we performed a systematic review and meta-analysis to evaluate the diagnostic accuracy of SPECT, PET, and MRS in differentiating PCNSL from other FBLs in HIV patients.

In our meta-analysis, SPECT had a pooled sensitivity of 0.92 and a specificity of 0.84. We found moderate heterogeneity in both sensitivity and specificity meta-analysis for SPECT. Our meta-regression analysis demonstrated that the analytical method used contributed to the heterogeneity in sensitivity. Specifically, studies using a quantitative approach tended to have higher sensitivity than those using a qualitative approach. This may reflect the decreased subjectivity and higher precision associated with computer automation.^[44]^ Another factor that contributed to the heterogeneity was the reference standard used. Most studies applied a combination of patient follow-up in addition to pathology and/or serology as their reference standard in determining the final diagnosis. We found that studies which used only pathology and/or serology as their gold standard had lower sensitivity and specificity than those which included patient follow-up as a reference standard in addition to pathology and/or serology. This suggests that the actual sensitivity and specificity of SPECT may be lower than expected. More studies using only pathology and/or serology as the gold standard are needed to reach a definite conclusion. Other factors that have been reported in the literature to further increase the sensitivity and/or specificity of SPECT include delayed imaging,^[45]^ 99Tcm-sestamibi as tracer instead of ^201^TI,^[15]^ larger lesion size,^[46]^ and the addition of serum Toxoplasma IgG^[47]^ and EBV PCR.^[48]^ Giancola et al suggested that HAART may artificially increase ^201^TI uptake in patients with toxoplasmosis, resulting in lower diagnostic accuracy in the post-HAART era.^[49]^ We could not compare the pooled sensitivity and specificity between pre- and post-HAART studies in the present study, since only 3 studies were published in the pre-HAART era.

PET may have higher sensitivity and specificity than SPECT. All 6 studies reported a sensitivity of 100%, while 4 out of 6 studies reported a specificity of 100%. However, the small number of studies and the lack of any false negatives in any of the 6 studies prevented meta-analysis.^[50]^ Although PET allows rapid evaluation of the whole body, and can detect the primary lesion of brain metastasis, which can mimic PCNSL,^[21]^ it has not been approved for this clinical indication due to limited supporting clinical data.^[51]^ Additionally, PET is more expensive than SPECT and somewhat less accessible (especially worldwide).

Other imaging modalities such as CT and MRI have also been studied for their ability to differentiate PCNSL from other contrast-enhancing lesions in HIV patients. However, routine CT and MRI demonstrated only modest sensitivity and specificity.^[5–10]^ In the present study, we included only 3 papers on MRS which contain extractable data, 2 of which reported only modest sensitivity and specificity in differentiating of lymphoma from other FBLs in HIV patients.^[31,32,35]^ Other advanced MRI techniques, such as apparent diffusion coefficient (ADC) ratios, and regional cerebral blood volume (rCBV) have even less reported evidence.^[34,52]^ According to our literature research, only 3 studies on diffusion^[26,27,34]^ and 1 study on MR perfusion^[28]^ were published on distinguishing lymphoma from other FBLs in HIV-infected patients. Two studies on diffusion demonstrated significant overlap in ADC ratios of toxoplasmosis and lymphoma.^[26,27]^ The only study on MR perfusion reported both sensitivity and specificity of 100% in distinguishing lymphoma from other FBLs in 13 patients.^[28]^ Further studies are need to investigate the diagnostic accuracy of these advanced MRI techniques.

We acknowledge several limitations of our study. First, some patients were under antitoxoplasmosis treatment when SPECT or PET was performed. Steroids and other drugs used can affect lymphoma presentations and complicate the differentiation of PCNSL from toxoplasmosis.^[14]^ Second, the SPECT tracer 201-Tl and PET tracer ^18^F-fluorodeoxyglucose can accumulate in abscesses and inflammatory lesions, which can increase false-positive rates and interfere with diagnosis of dual pathology.^[13]^ Third, small sample size, retrospective design, heterogeneous tumor characteristics, and different imaging parameters such as scanning time can contribute to bias in estimating diagnostic accuracy of SPECT, PET, and MRS. Fourth, lesion size was reported in a small number of studies, which precluded analysis of diagnostic accuracy based on a lesion size threshold. Finally, there was evidence of publication bias in our meta-analysis of SPECT, which suggests that inclusion of small studies may have skewed our estimates for test accuracy. However, such bias is not very concerning in meta-analysis of single outcome proportions because no effect estimate is calculated (ie, no comparison between groups is made).

## 5 Conclusion

SPECT has good diagnostic accuracy in differentiating PCNSL from other FBLs in HIV patients, but the actual sensitivity and specificity may be lower than expected when only pathology and/or serology was used as the gold standard. PET may be superior but has less supporting clinical data and is more expensive.

## Supplementary Material

Supplemental Digital Content

## Supplementary Material

Supplemental Digital Content
