# Supplementary material for: Diagnostic accuracy of SPECT, PET, and MRS for primary central nervous system lymphoma in HIV patients: A systematic review and meta-analysis
Source: Medicine (Baltimore). 2017 May 12;96(19):e6676. doi: 10.1097/MD.0000000000006676 (PMC5428578; doi:10.1097/MD.0000000000006676)
Supplement: Supplemental Digital Content [file medi-96-e6676-s001.doc]

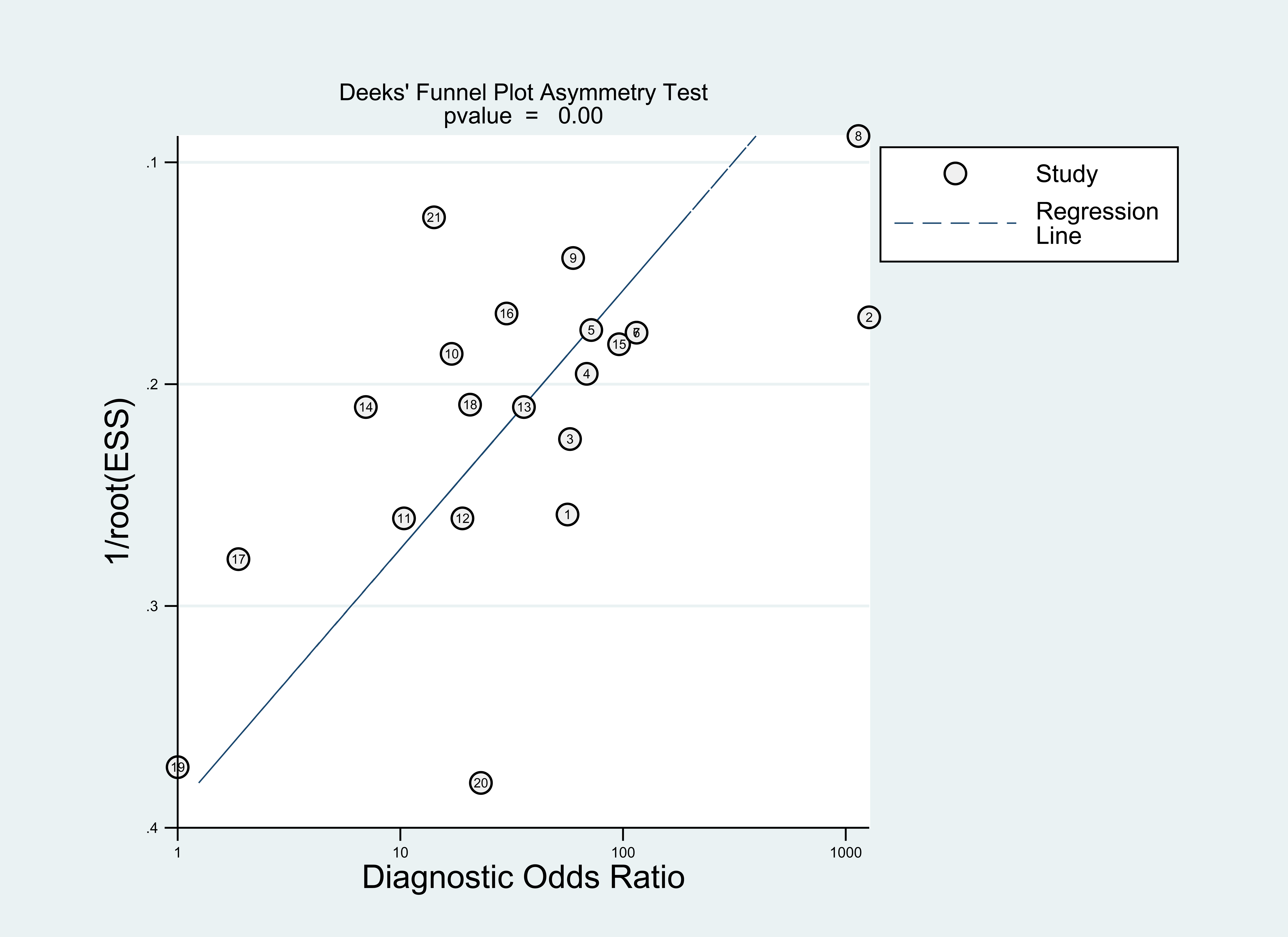


Supplem Figure 1: Funnel plot for Deeks' test based on the data of SPECT for differentiating lymphoma from non-lymphoma focal brain lesions in HIV. Note: ESS: effective sample size.
