# Supplementary material for: Diagnostic accuracy of SPECT, PET, and MRS for primary central nervous system lymphoma in HIV patients: A systematic review and meta-analysis
Source: Medicine (Baltimore). 2017 May 12;96(19):e6676. doi: 10.1097/MD.0000000000006676 (PMC5428578; doi:10.1097/MD.0000000000006676)
Supplement: Supplemental Digital Content [file medi-96-e6676-s002.pdf]

Supplemental Table 1: study and patient characteristics of eligible SPECT studies

study	Year of publication	Analytical method	Number of patients	Mean age (age range)(y)	Imaging method	tracer	Study design	Country	Reference standard	Final non-lymphoma diagnoses
O'Malley et al.[16]	1994	quantitative	13	34(26-47)	SPECT	201-Tl	prospective	USA	pathologiy, follow up	5 TE,1 PML,1 venous angioma
Ruiz et al.[17]	1994	visual	37	34(26-46)	SPECT	201-Tl	prospective	USA	pathology, serology, follow up	24 TE,1 mycobacterium tuberculosis abscess
Lorberboym et al.[14]	1996	quantitative	18	39(29-53)	SPECT	201-Tl	prospective	USA	pathology, serology, follow up	8 TE,1 crytococcosis,1 metastatic adenocarcinoma
D'Amico et al.[24]	1997	quantitative	37	34.7(25-64)	SPECT	201-Tl	prospective	Italy	pathology, follow up	24 TE,1 TB
Castagna et al.[11]	1997	quantitative	27	NA	SPECT	201-Tl	prospective	Italy	pathology, serology	17 TE, 1 TB
Miller et al.[10]	1998	quantitative	30	36(27-55)	SPECT	201-Tl	retrospective	UK	pathology, serology, follow up	NA
Kessler et al.[41]	1998	quantitative	162	NA	SPECT	201-Tl	retrospective	USA	pathology, serology, follow up	NA
Shyam babu et al.[5]	2012	visual	18	38.3(16-58)	SPECT	99m-Tc	prospective	India	pathology, serology, follow up	15 TE, 1 PML, 1 encephalitis (probable tuberculoma)
Sakamoto et al.[51]	2014	visual	13	41(33-62)	SPECT	201-Tl	retrospective	Japan	pathology, serology, follow up	11 TE, 1 TE and crytococcosis
Hussain et al.[45]	2016	visual	68	NA	SPECT	201-Tl	retrospective	USA	pathology	NA
Lorberboym et al.[42]	1998	quantitative	49	39(26-53)	SPECT	201-Tl	prospective	USA	pathology, follow up	22 TE, 1 TB,1 metastactic adenocarcinoma, 1 crytococcosis, 1 cerebral gliosis
DeLaPena et al.[37]	1998	quantitative	36	33.2(21-47)	SPECT	201-Tl, 99m-Tc	prospective	USA	pathology, follow up	16 TE, 1 PML, 1 abscess, 1 microabscesses, 1 gliosis and chronic inflammation, 2 demyelinating process, 1 Candida tropicalis, 1 subacute encephalitis
Antinori et al.[13]	1999	visual	31	38	SPECT	201-Tl	prospective	Italy	pathology, serology, follow up	10 TE, 1 PML,3 CMV encephalitis, 1 VZV encephalitis, 1 crytococcosis,1 CNS vasculitis,1 TB
Lee et al.[50]	1999	NA	21	37(28-72)	SPECT	201-Tl,67-Gl	retrospective	USA	pathology	1 astrocytoma, 1 glioblastoma, 3 PML, 5 infection, 1 infarct, 1 hemorrhage

Skiest et al.[44]	2000	visual	38	35	SPECT	201-Tl	retrospective	USA	pathology, serology, follow up	17 TE, 3 PML, 1 TB, 1 aspergillus, 1 crytococcosis, 1 varicella-zoster virus
Licho et al.[25]	2002	visual	14	(28-55)	SPECT	201-Tl	prospective	USA	pathology	5 TE, 1 PML, 1 HSV, 2 gliosis
Naddaf et al.[15]	1998	quantitative	17	NA	SPECT	201-Tl, 99m-Tc	prospective	USA	pathology, serology, follow up	9 TE,2 PML,1 necrosis,1 haemorrhage
Giancola et al.[46]	2004	quantitative	38	41.5(36-45)	SPECT	201-Tl	prospective	Italy	serology	19 TE, 4 EUO, 5 PML, 1 TB, 1 metastatic lesion, 1 cryptococcosis

note:-NA indicates the data not available; 201-Tl, thallium-201 chloride; 99m-Tc, technetium sestamibi; 67-Gl, gallium-67; TE, toxoplasma encephalitis; PML, progressive multifocal leukoencephalopathy; TB tuberculosis; HSV, herpes simplex virus; EUO, encephalopathy of unknown origin.

Supplemental Table 2: study and patient characteristics of eligible PET studies

study	Year of publication	Analytical method	Number of patients	Mean age (age range)(y)	Imaging method	tracer	Country	Study design	Reference standard	Final non-lymphoma diagnoses
Lewitschnig et al.[20]	2013	visual	29	47(25-79)	PET	18F-FDG	UK	retrospective	pathology, follow-up	10 TE, 1 PML, 1 non-small cell lung cancer, 12 infectious diseases
Pierce et al.[22]	1995	visual	17	35.9(25-50)	PET	18F-FDG	USA	prospective	pathology, serology, follow-up	8 T,2 PML,1 crytococcosis
Westwood et al.[36]	2013	visual	10	40(25-74)	PET	18F-FDG	UK	prospective	pathology, follow-up	6 TE,1 lung cancer metastasis, 1 PML
Villinger et al.[23]	1995	quantitative	11	39(27-57)	PET	18F-FDG	Germany	prospective	pathology, follow-up	6 TE, 1 TB
Heald et al.[18]	1996	quantitative	18	38.7(23-66)	PET	18F-FDG	USA	prospective	pathology, serology, follow-up	4 TE, 2PML, 2 syphilis
O'Doherty et al.[21]	1997	quantitative	23	29.6(17-53)	PET	18F-FDG	UK	prospective	pathology, follow-up	13 TE, 3 PML, 1 oligodendroglioma

note:-NA indicates the data not available; 18F-FDG, 18F-fluorodeoxyglucose; TE, toxoplasmic encephalitis; PML, progressive multifocal leukoencephalopathy; TB, tuberculosis.

Supplemental Table 3: study and patient characteristics of eligible MRS studies

study	Year Of publication	Number of patients	Mean age (age range)(y)	Imaging method	Country	Study design	Analytical method	Reference standard	Final non-lymphoma diagnoses
Westwood et al.[36]	2013	10	40(25-74)	MRS	UK	prospective	Visual	pathology, follow-up	6 TE,1 lung cancer metastasis, 1 PML
Simone et al.[36]	1998	60	32.4(16-64)	MRS	Italy	prospective	quantitative	pathology, follow-up	20 TE, 8PML, 25 HIV-related encephalopathies
Chinn et al.[32]	1995	26	39.6(28.8-58)	MRS	UK	prospective	quantitative	pathology,serology follow-up	18 TE

note: TE, toxoplasma encephalitis; PML, progressive multifocal leukoencephalopathy.

Supplemental Table 4: Quality assessment for 26 articles (QUADAS-2)

Study	RISK OF BIAS				APPLICABILITY CONCERNS		
	PATIENT SELECTION	INDEX TEST	REFERENCE STANDARD	FLOW AND TIMING	PATIENT SELECTION	INDEX TEST	REFERENCE STANDARD
Babu et al.[5]	H	L	L	L	H	L	L
Licho et al.[25]	L	H	L	H	L	L	L
Hussain et al.[45]	L	L	L	L	L	L	L
Skiest et al.[44]	L	H	L	H	L	L	L
Antinori et al.[13]	L	H	L	L	L	L	L
Naddaf et al.[15]	L	H	L	L	L	L	L
Miller et al.[10]	L	U	L	L	L	L	L
DeLaPena et al.[37]	L	L	L	H	L	L	L
Lorberboym et al.[14]	L	U	L	L	H	L	L
Kessler et al.[41]	L	L	L	L	L	L	L
DAmico et al.[24]	L	H	L	L	L	L	L
Lorberboym et al.[42]	L	U	L	L	H	L	L
Ruiz et al.[17]	L	L	L	L	L	L	L
O'Malley et al.[16]	L	L	L	H	L	L	L
Giancola et al.[46]	L	L	L	L	L	L	L
Sakamoto et al.[51]	L	L	U	L	L	L	U

Castagna et al.[11]	L	L	L	L	L	L	L
Lee et al.[50]	L	U	L	L	L	U	L
Lewitschnig et al.[20]	L	U	L	L	L	L	L
Pierce et al.[22]	L	L	L	H	L	L	L
Westwood et al.[36]	L	L	L	L	H	L	L
Villinger et al.[25]	H	L	L	L	H	L	L
Heald et al.[28]	H	H	L	L	H	L	L
ODoherty et al.[21]	L	L	L	L	H	L	L
Simone et al.[36]	L	L	L	L	H	L	L
Chinn et al.[32]	L	L	L	H	L	L	L

note: L, Low Risk; H, High Risk; U, Unclear Risk.

Online Table 5: Influence of individual studies for diagnostic performance of SPECT in CNS lymphomas

			Comparison with Overall (P Value)	
**Excluding Study One by One**	**Pooled Sensitivity (95% CI)**	**Pooled Specificity (95% CI)**	**Sensitivity**	**Specificity**
Overall	0.92 (0.85 - 0.96)	0.84 (0.74 - 0.90)		
Antinori et al. 1999	0.92 (0.84 - 0.96)	0.83 (0.73 - 0.90)	>0.05	>0.05
Babu et al. 2013	0.92 (0.85 - 0.96)	0.84 (0.74 - 0.91)	>0.05	>0.05
Castagna et al. 1997	0.92 (0.85 - 0.96)	0.82 (0.73 - 0.89)	>0.05	>0.05
D'Amico et al. 1997	0.92 (0.85 - 0.96)	0.83 (0.73 - 0.89)	>0.05	>0.05
DeLaPena et al. 1998	0.92 (0.85 - 0.96)	0.83 (0.73 - 0.89)	>0.05	>0.05
DeLaPena et al. 1998	0.92 (0.85 - 0.96)	0.83 (0.73 - 0.89)	>0.05	>0.05
Giancola et al. 2004	0.92 (0.84- 0.96)	0.84 (0.74 - 0.91)	>0.05	>0.05
Hussain et al. 2016	0.93 (0.86 - 0.96)	0.84 (0.74 - 0.91)	>0.05	>0.05
Kessler et al. 1998	0.89 (0.82 - 0.94)	0.82 (0.73 - 0.89)	>0.05	>0.05
Lee et al. 1999	0.91 (0.84 - 0.95)	0.85 (0.78 - 0.91)	>0.05	>0.05
Lee et al. 1999	0.91 (0.84 - 0.95)	0.84 (0.75 - 0.91)	>0.05	>0.05
Licho et al. 2002	0.93 (0.86 - 0.96)	0.85 (0.75 - 0.91)	>0.05	>0.05
Lorberboym et al. 1996	0.91 (0.84 - 0.95)	0.84 (0.74 - 0.91)	>0.05	>0.05
Lorberboym et al. 1998	0.91 (0.83 - 0.96)	0.84 (0.75 - 0.91)	>0.05	>0.05
Miller et al. 1998	0.92 (0.84 - 0.96)	0.85 (0.76 - 0.91)	>0.05	>0.05
Naddaf et al. 1998	0.91 (0.84 - 0.95)	0.85 (0.76 - 0.91)	>0.05	>0.05
Naddaf et al. 1998	0.91 (0.84 - 0.95)	0.84 (0.75 - 0.91)	>0.05	>0.05
O'Malley et al. 1994	0.91 (0.84 - 0.95)	0.84 (0.74 - 0.90)	>0.05	>0.05
Ruiz et al. 1994	0.91 (0.84 - 0.95)	0.82 (0.73 - 0.89)	>0.05	>0.05
Sakamoto et al. 2014	0.92 (0.84 - 0.96)	0.83 (0.73 - 0.90)	>0.05	>0.05

Skiest et al. 2000	0.92 (0.85 - 0.96)	0.84 (0.74 - 0.90)	>0.05	>0.05

note: DeLaPena et al., Lee et al., Naddaf et al. applied two tracers for each single study.
